# Effective isopropanol–butanol (IB) fermentation with high butanol content using a newly isolated *Clostridium* sp. A1424

**DOI:** 10.1186/s13068-016-0650-7

**Published:** 2016-10-26

**Authors:** Sung Hun Youn, Kyung Min Lee, Ki-Yeon Kim, Sun-Mi Lee, Han Min Woo, Youngsoon Um

**Affiliations:** 1 Clean Energy Research Center, Korea Institute of Science and Technology (KIST), Hwarangno 14‑gil 5, Seongbuk‑gu, Seoul, 02792 South Korea; 2 Clean Energy and Chemical Engineering, Korea University of Science and Technology, 217 Gajeong‑ro, Yuseong‑gu, Daejeon, 34113 South Korea; 3 Department of Food Science and Biotechnology, Sungkyunkwan University (SKKU), 2066 Seobu-ro, Jangan-gu, Suwon, 16419 South Korea

**Keywords:** *Clostridium* sp. A1424, Butanol, Isopropanol, Mixture of glucose and glycerol, Crude glycerol

## Abstract

**Background:**

Acetone–butanol–ethanol fermentation has been studied for butanol production. Alternatively, to achieve acetone-free butanol production, use of clostridium strains producing butanol and 1,3-propanediol (1,3-PDO) from glycerol, natural and engineered isopropanol–butanol–ethanol (IBE) producers has been attempted; however, residual 1,3-PDO and acetone, low IBE production by natural IBE producers, and complicated gene modification are limitations.

**Results:**

Here, we report an effective isopropanol and butanol (IB) fermentation using a newly isolated *Clostridium* sp. A1424 capable of producing IB from various substrates with a small residual acetone. Notably, this strain also utilized glycerol and produced butanol and 1,3-PDO. After 46.35 g/L of glucose consumption at pH 5.5-controlled batch fermentation, *Clostridium* sp. A1424 produced 9.43 g/L of butanol and 13.92 g/L of IB at the productivity of 0.29 and 0.44 g/L/h, respectively, which are the highest values in glucose-based batch fermentations using natural IB producers. More interestingly, using glucose–glycerol mixtures at ratios ranging from 20:2 to 14:8 led to not only acetone-free and 1,3-PDO-free IB fermentation but also enhanced IB production along with a much higher butanol content (butanol/isopropanol ratio of 1.81 with glucose vs. 2.07–6.14 with glucose–glycerol mixture). Furthermore, when the mixture of glucose and crude glycerol at the ratio of 14:8 (total concentration of 35.68 g/L) was used, high butanol/isopropanol ratio (3.44) and butanol titer (9.86 g/L) were achieved with 1.4-fold enhanced butanol yield (0.28 g/g) and productivity (0.41 g/L/h) compared to those with glucose only at pH 5.5.

**Conclusions:**

A newly isolated *Clostridium* sp. A1424 was able to produce butanol and isopropanol from various carbon sources. The productivity and titer of butanol and total alcohol obtained in this study were higher than the previously reported results obtained using other natural IB producers. Use of the mixture of glucose and glycerol was successful to achieve acetone-free, 1,3-PDO-free, and enhanced IB production with higher yield, productivity, and selectivity of butanol compared to those with glucose only, providing great advantages from the perspective of carbon recovery to alcohols. This notable result could be accomplished by isolating an effective IB producer *Clostridium* sp. A1424 as well as by utilizing glucose–glycerol mixtures.

**Electronic supplementary material:**

The online version of this article (doi:10.1186/s13068-016-0650-7) contains supplementary material, which is available to authorized users.

## Background

The C_4_ alcohol *n*-butanol is broadly used as an intermediate of pharmaceuticals and polymers; an extractant of antibiotics, hormones, and vitamins; and a swelling agent in textiles [1, 2]. Until now, it has been industrially produced through petrochemical processes [3]. Bio-butanol production was started in the 1900s via acetone–butanol–ethanol (ABE) fermentation using clostridia. The main aim of this process, particularly during the periods of the first and second world wars, was the production of acetone for manufacturing cordite [4]. As bio-butanol has received great attention because of its suitable properties as an alternative and renewable fuel [3], the efficient production of bio-butanol has been widely studied through the bioprocess engineering and the strain development [2, 5, 6]. Acetone accounting for 20–30% of ABE production is considered as an undesirable product because of its corrosiveness and poor fuel properties [1]. For this reason, there has been much effort to reduce or eliminate the production of acetone by interrupting the acetone pathway using genetic manipulation. However, disruption of the genes responsible for acetone formation led not only to less acetone production but also decreased butanol production with the accumulation of acetic acid, butyric acid, or ethanol [7–9].

Alternatively, butanol production has been investigated with non-acetone producing *Clostridium pasteurianum*, which is capable of producing butanol and 1,3-propanediol (1,3-PDO) from glycerol [10–14]. Crude glycerol generated from biodiesel manufacture has emerged as a low cost substrate generating a high level of the reducing equivalents required for the production of reduced metabolites such as butanol [2]. Although *C. pasteurianum* is different from other butanol-producing clostridia in terms of utilizing glycerol as a sole carbon source for butanol production, it also produces 1,3-PDO as a major byproduct, instead of acetone. Attempts have been made to decrease the production of 1,3-PDO using a chemically mutated strain [15] and using a mixture of glucose and glycerol as substrates [16, 17]; but a certain amount of 1,3-propanediol was still produced.

Butanol can also be produced through isopropanol–butanol–ethanol (IBE) fermentation with a small residual amount of acetone by wild-type strains [18–20] and engineered strains [1, 21, 22]. Isopropanol in the mixed alcohols can be utilized as a fuel additive [20]. However, there are only a few natural IBE producers reported to date, including *Clostridium beijerinckii* NRRL B-593 [19, 20] and *Clostridium beijerinckii optinoii* [18], and they show low production of butanol and alcohols even after process optimization [18, 20, 23]. Another approach is to genetically engineer ABE strains by expressing the isopropanol dehydrogenase from *C. beijerinckii* NRRL B-593 [1, 21, 22]. However, overexpression of isopropanol dehydrogenase alone caused an incomplete conversion of acetone to isopropanol and decrease of butanol production [1, 21, 22, 24]. Multiple genes had to be modified to restore butanol production [1, 24, 25]. Because genetic modification of solventogenic clostridia is far more challenging than for other better known microorganisms, isolating superior new natural IBE producers would widen the feasibility of efficient IBE fermentation.

Here, we report a newly isolated *Clostridium* sp. A1424 which is able to perform isopropanol–butanol (IB) fermentation along with a small amount of acetone using glucose. Unlike other solventogenic clostridia, this strain utilizes glycerol as the sole carbon source and produces butanol and 1,3-PDO as a byproduct. The effects of carbon source, glucose concentration, and pH on IB production of *Clostridium* sp. A1424 were evaluated. Notably, using a mixture of glucose and glycerol resulted in (i) enhanced IB production, (ii) much higher butanol contents, (iii) no residual acetone, and (iv) no 1,3-PDO production even in the presence of glycerol. This is the first report of success in increasing butanol content and decreasing isopropanol production simultaneously in IB fermentation. The results presented here suggest that the use of the high IB producing strain *Clostridium* sp. A1424 and process optimization would be a good strategy for industrial application of IB fermentation towards higher butanol content.

## Results and discussion

### Identification of *Clostridium* sp. A1424

**Fig. 1:**
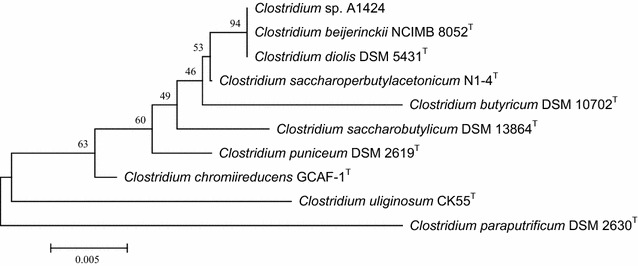
Phylogenetic tree with *Clostridium* sp. A1424 and its closely related type strains based on 16S rRNA gene sequences

A total of six colonies producing butanol were obtained from the soil samples. Among them, a bacterium producing butanol and isopropanol from glucose was isolated. Based on its 16S rRNA sequence and phylogenetic analysis, the isolate was found to belong to the genus *Clostridium* and to be very closely related to *C. beijerinckii* NCIMB 8052^T^ and *Clostridium diolis* DSM 5431^T^ (Fig. 1), with almost identical 16S rRNA sequence similarity (99.92%) to both strains (Table 1), and was designated *Clostridium* sp. A1424. However, this strain was distinguished from *C. beijerinckii* NCIMB 8052^T^ and *C. diolis* DSM 5431^T^ by its physiological differences in metabolite production from glucose and glycerol (Table 1). *Clostridium beijerinckii* NCIMB 8052^T^ produces butanol and acetone as the main products using glucose, but it cannot utilize glycerol as a sole carbon source [26, 27]. *Clostridium diolis* DSM 5431^T^ mainly produces butyric acid and acetic acid from glucose and it can utilize glycerol, yielding 1,3-PDO [28, 29]. On the other hand, *Clostridium* sp. A1424 showed significantly different features: it mainly produced butanol and isopropanol from glucose and butanol and 1,3-PDO from glycerol. The fermentation products of non-type strains of solventogenic clostridia including natural IB producers were also compared with those of *Clostridium* sp. A1424 in Table 1. When glycerol is supplied as a sole carbon source, *C. beijerinckii* NRRL B-593 mainly produces 1,3-PDO and a small amount of ethanol and 2,3-butanediol [30]. Study on the glycerol utilization of *C. beijerinckii optinoii* has not yet been reported and *C. pasteurianum* DSM 525 produces acids as the main products from glucose [12]. Because *Clostridium* sp. A1424 was able to produce butanol as the main product from both glucose and glycerol and to produce isopropanol from glucose, we further investigated *Clostridium* sp. A1424 as a potential butanol and isopropanol producer.

**Table 1:** Comparison of fermentation products from glucose and glycerol between *Clostridium* sp. A1424 and other related clostridia

Strains	GenBank accession no.	16s rRNA similarity (%)	Substrate	Main products	References
*Clostridium* sp. A1424	KT314078	100	Glucose	Butanol, isopropanol	This study
			Glycerol	Butanol, 1,3-propanediol	
*C. beijerinckii* NCIMB 8052^T^	CP000721	99.92	Glucose	Butanol, acetone	[26, 27]
			Glycerol	–	
*C. diolis* DSM 5431^T^	AJ458418	99.92	Glucose	Butyric acid, acetic acid	[28, 29]
			Glycerol	1,3-Propanediol	
*C. beijerinckii* NRRL B-593^a^	U16168.1	99.82^a^	Glucose	Butanol, isopropanol	[20, 30]
			Glycerol	1,3-Propanediol, ethanol	
*C. beijerinckii optinoii*	N/A	N/A	Glucose	Butanol, isopropanol	[18]
			Glycerol	N/A	
*C. pasteurianum* DSM 525^T^	NR_104822.1	92.41	Glucose	Butyric acid, acetic acid	[12, 14, 17]
			Glycerol	Butanol, 1,3-propanediol	

*N/A* not available

^a^16S rRNA sequence of *C. beijerinckii* NRRL B-593 consisting of 550 bp (NCBI Genbank: U16168.1) was used. Other sequences were about 1330 bp

### Characterization of *Clostridium* sp. A1424: substrate utilization and metabolite production

For evaluating carbon source utilization and metabolite production of *Clostridium* sp. A1424, various carbon sources including hexose (glucose, mannose, fructose, and galactose), pentose (xylose and arabinose), disaccharides (cellobiose and sucrose), and glycerol were tested.

**Fig. 2:**
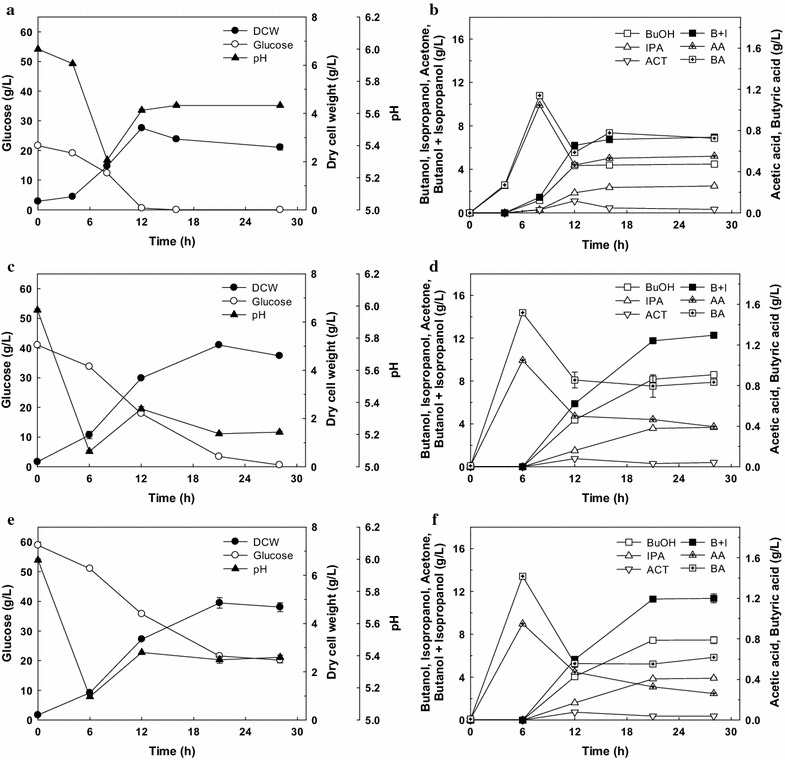
Isopropanol–butanol fermentation with 21.6 g/L initial glucose concentration (**a**, **b**), 41.0 g/L initial glucose concentration (**c**, **d**), and 58.9 g/L initial glucose concentration (**e**, **f**). *DCW* dry cell weight, *BuOH* butanol, *IPA* isopropanol, *ACT* acetone, *B* *+* *I* butanol + isopropanol, *AA* acetic acid, *BA* butyric acid. The fermentation was performed on serum bottles without pH control. *Error bars* represent one standard deviation of triplicate experiments

*Clostridium* sp. A1424 utilized all the tested substrates with varying extent of consumption. Interestingly, *Clostridium* sp. A1424 exhibited distinct metabolite production patterns depending on carbon sources (Table 2). Glucose, mannose, fructose, and disaccharides (18–22 g/L) were completely consumed and the main products were butanol and isopropanol, with butanol yields (*Y*_B_) of 0.21–0.28 g/g substrate. The sum of butanol and isopropanol yield (*Y*_IB_) was 0.31–0.37 g/g substrate. The yields are comparable with those of other natural IBE producers [18, 20]. Fermentation with sucrose showed the highest concentration and yield of butanol among the tested substrates. The fermentation profiles of *Clostridium* sp. A1424 with those substrates were typical of normal solventogenic fermentation (see Fig. 2a for glucose cultures as a representative example). Butyric acid and acetic acid were produced with pH drop (i.e., acidogenic phase), and then butanol, isopropanol, and acetone concentration increased by consuming organic acids with pH rise (i.e., solventogenic phase). The acetone concentration decreased later with time as acetone was likely further converted to isopropanol by isopropanol dehydrogenase with NADPH as a co-factor [1]. On the other hand, in the case of galactose, xylose, and arabinose, *Clostridium* sp. A1424 mainly produced butyric acid with relatively low substrate consumption and acidogenic pH profiles (see Additional file 1: Figure S1 for xylose cultures as a representative example). Considering that acetone and isopropanol were not detected in the galactose, xylose, and arabinose cultures, it appeared that acid re-assimilation by CoA transferase [1] might not be triggered. Interestingly, when exogenous acetone was initially added to xylose cultures, acetone was readily converted to isopropanol from the beginning of fermentation (Additional file 2: Figure S2). This result indicates that the co-factor for isopropanol dehydrogenase (e.g., NADPH) was not limiting in these conditions and that isopropanol dehydrogenase was expressed independently to ABE production-related enzymes that are known such as CoA transferase, alcohol/aldehyde dehydrogenase, and acetoacetate decarboxylase. These are known to be induced together during the solventogenic phase [1].

**Table 2:** Fermentation products of *Clostridium* sp. A1424 using various substrates

Substrate	Substrate consumed (g/L)	Products (g/L)						Yield (g/g)	
		Butanol	Isopropanol	Acetone	Acetic acid	Butyric acid	1,3-propanediol	*Y*_B_^a^	*Y*_IB_^a^
Glucose	21.62_±0.06_	4.49_±0.04_	2.48_±0.06_	0.33_±0.03_	0.43_±0.06_	0.72_±0.02_	0	0.21	0.32
Mannose	19.92_±0.04_	4.40_±0.13_	1.88_±0.01_	0.33_±0.04_	0.66_±0.02_	0.93_±0.05_	0	0.24	0.34
Fructose	21.63_±0.05_	4.38_±0.02_	1.63_±0.06_	0.19_±0.01_	0.85_±0.01_	1.49_±0.03_	0	0.23	0.31
Galactose	5.96_±0.14_	0.59_±0.01_	0	0	0.44_±0.02_	1.90_±0.02_	0	0.11	0.11
Xylose	12.81_±0.09_	0.87_±0.03_	0	0	0.68_±0.02_	4.71_±0.00_	0	0.08	0.08
Arabinose	8.24_±0.04_	0	0	0	0.15_±0.01_	4.20_±0.05_	0	0	0
Cellobiose	20.02_±0.04_	4.50_±0.02_	2.65_±0.00_	0.45_±0.01_	0.43_±0.01_	0.24_±0.01_	0	0.23	0.37
Sucrose	18.20_±1.13_	5.01_±0.04_	1.71_±0.02_	0.34_±0.02_	0.97_±0.00_	0.87_±0.07_	0	0.28	0.37
Glycerol	18.13_±1.10_	3.91_±0.19_	0	0	0.27_±0.02_	2.20_±0.07_	3.40_±0.25_	0.23	0.23

The initial substrate concentration was 18–22 g/L for each substrate

The fermentation was performed on serum bottle without pH control

The data were obtained at 28 h of fermentation except sucrose cultures (36 h)

The values are average and one standard deviation of triplicate experiments

^a^ *Y*_B_, butanol yield (g butanol/g substrate); *Y*_IB_, the sum of butanol and isopropanol yield (g [butanol + isopropanol]/g substrate)

When glycerol was used as a substrate, the main products were butanol and 1,3-PDO, with butanol yield of 0.23 g/g (*Y*_B_). To date, *C. pasteurianum* is the only natural butanol producer from glycerol as sole carbon source known [2] with a yield (*Y*_B_) of 0.25–0.30 g/g [11, 12, 14, 17].

To date, two wild-type clostridia strains, *C. beijerinckii* NRRL B-593 [20] and *C. beijerinckii optinoii* [18], have already been studied for butanol and isopropanol production; however, there is limited information on the carbon utilization ability and metabolite production patterns of those strains. In this study, *Clostridium* sp. A1424 was shown to consume various carbon sources. Of interest was that *Clostridium* sp. A1424 performed IB fermentation or butyric acid-producing fermentation depending on carbon source (Table 2). However, according to preliminary experiments in our lab, supplying varied ratios of mixed sugars with glucose (yielding IB production) and xylose (yielding butyric acid production) to *Clostridium* sp. A1424 also led to efficient production of butanol and isopropanol (*Y*_IB_ = 0.30–0.34 g/g of total sugar, data not shown). Further study on IB production with mixed sugars derived from lignocellulosic biomass will be conducted to elucidate metabolic regulation in *Clostridium* sp. A1424.

### Effect of glucose concentration on the production of butanol and isopropanol

Further fermentation studies using serum bottles were performed with 20–60 g/L of initial glucose as sole carbon source to investigate the extent of glucose utilization and solvent production.

As shown in Fig. 2a, b, 4.41 g/L of butanol and 2.35 g/L of isopropanol were the main products after consumption of 21.62 g/L glucose after 16 h with butanol yield (*Y*_B_) of 0.20 g/g and overall butanol productivity of 0.28 g/L/h. When the initial glucose concentration was increased to 41.02 g/L, butanol and isopropanol concentrations were also increased to 8.60 and 3.68 g/L, respectively, with butanol yield of 0.21 g/g and butanol productivity of 0.31 g/L/h at 28 h (Fig. 2c, d). With the initial glucose concentration of 58.90 g/L, the production of total alcohol showed no further increase (butanol, 7.47 g/L; isopropanol, 3.90 g/L) with incomplete glucose consumption (38.73 g/L) (Fig. 2e, f).

Overall, the butanol yield (*Y*_B_) and the butanol productivity (*P*_B_) were 0.19–0.21 g/g and 0.27–0.31 g/L/h, respectively. In the case of total alcohol (i.e., butanol + isopropanol), the yield (*Y*_IB_) and the productivity (*P*_IB_) were 0.29–0.31 g/g and 0.41–0.44 g/L/h, respectively. Although these results could not be directly compared with other reports employing different cultivation conditions, the concentration and productivity of alcohols by *Clostridium* sp. A1424 are higher than those of other natural IBE producers in batch fermentation; *C. beijerinckii* NRRL B-593 produced 3.71 and 2.16 g/L of butanol and isopropanol, respectively, after 48 h with low productivity (*P*_B_, 0.08 g/L/h; *P*_IB_, 0.12 g/L/h) [20], while *C. beijerinckii optinoii* was shown to produce 6.24 and 3.21 g/L of butanol and isopropanol, respectively, after 48 h (*P*_B_, 0.13 g/L/h; *P*_IB_, 0.20 g/L/h) [18].

*Clostridium* sp. A1424 appears to produce less butanol than well-known natural ABE producing clostridia such as *Clostridium acetobutylicum* and *C. beijerinckii* producing over 10 g/L of butanol [26, 31]. However, the butanol productivity obtained with *Clostridium* sp. A1424 is higher than that with ABE producing clostridia (0.17–0.23 g/L/h) [26, 31]. Even compared with the results for clostridia mutated and genetically modified to enhance butanol production in ABE fermentation (0.23–0.38 g/L/h) [5, 6, 32–34], *Clostridium* sp. A1424 shows comparable butanol productivity (*P*_B_). Considering that high butanol productivity likely enhances the butanol recovery rate for in situ butanol removal processes (e.g., gas stripping, adsorption) incorporated to reduce butanol inhibition [35], *Clostridium* sp. A1424 appears a good candidate for butanol production, and an even better one for concomitantly producing isopropanol instead of acetone.

### Effect of pH on butanol and isopropanol production

**Fig. 3:**
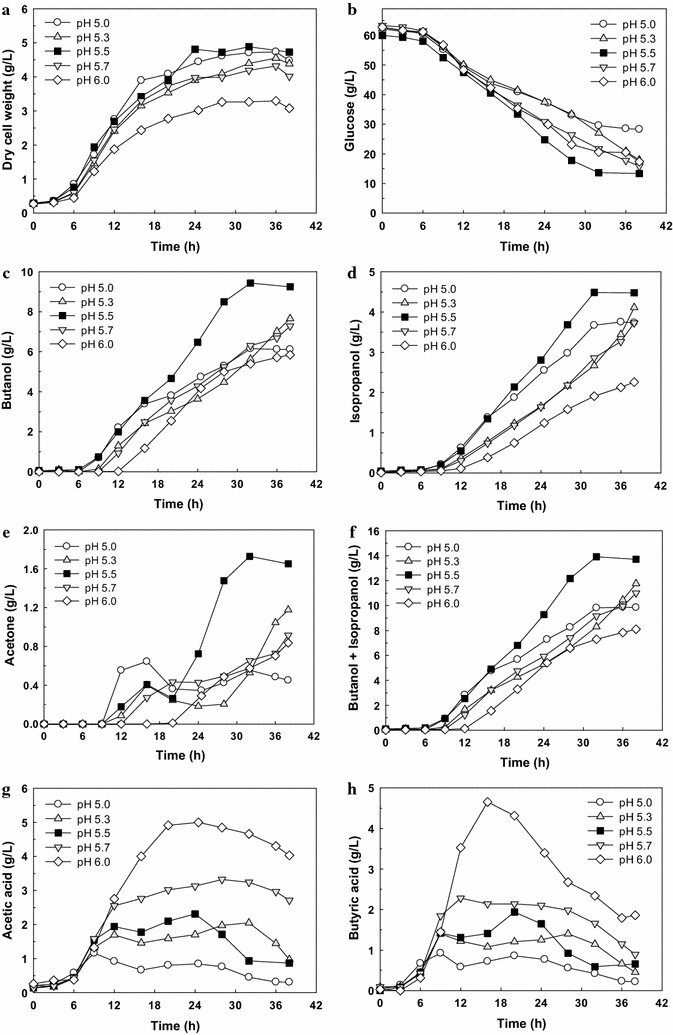
The pH-controlled fermentation of *Clostridium* sp. A1424. **a** Dry cell weight, **b** glucose, **c** butanol, **d** isopropanol, **e** acetone, **f** butanol and isopropanol, **g** acetic acid, and **h** butyric acid. The data are from a single run

As pH is known to be one of the key factors influencing ABE production [6], the effect of pH on the production profiles was investigated for *Clostridium* sp. A1424. The initial pH was around 6.0 and pH was maintained at a set point (6.0, 5.7, 5.5, 5.3, and 5.0) once the pH decreased to that point. Because glucose consumption during the pH-controlled fermentation was expected to be higher than that of no pH-controlled serum bottle test (Fig. 2), the initial glucose concentration was increased to 60 g/L for avoiding carbon source limitation. The cell growth (Fig. 3a) was similar to, or slightly higher than, that with no pH control (Fig. 2e) except for fermentation at pH 6.0 revealing much lower cell growth (Fig. 3a). Moreover, glucose consumption at pH 5.0 was lower than that of other pH-controlled cultures (Fig. 3b).

The best performance in alcohol production was obtained at pH 5.5 after 32 h (9.43 g/L butanol and 4.49 g/L isopropanol) from 46.35 g/L glucose. This result is superior to butanol and isopropanol production without pH control (shown in Fig. 2f) by 26.2 and 15.1%, respectively. The yield (*Y*_B_ and *Y*_IB_) and productivity (*P*_B_ and *P*_IB_) of butanol and total alcohol at pH 5.5 were 0.20 and 0.30 g/g, and 0.29 and 0.44 g/L/h, respectively. These results are similar to those obtained with serum bottle cultivation without pH control.

The balance between NAD(P)H and NAD(P)^+^ plays a key role in the production of solvents in clostridia. The generation of NAD(P)H in solventogenic clostridia occurs not only through the glycolytic pathway but also by the pyruvate ferredoxin oxidoreductase (PFOR) at the expense of hydrogen to produce net NAD(P)H-consuming products such as butanol (Additional file 3: Table S1) [13]. As shown in Additional file 4: Figure S3, the total required NAD(P)H for metabolite production at pH 5.0–5.5 was higher than NAD(P)H from the glycolytic pathway, indicating the additional NAD(P)H generation by PFOR through the oxidation of reduced ferredoxin (Fd_red_). Especially, among the results at pH 5.3–6.0 revealing a similar glucose consumption, the amount of NAD(P)H generated from Fd_red_ at pH 5.5 accounted for up to 12.0% of the total required NAD(P)H, agreeing with the best performance in alcohol production. On the other hand, the highest level of acetone was also obtained at pH 5.5. It seems that the generated NAD(P)H was not sufficient for the conversion of acetone to isopropanol because of a high demand of NAD(P)H in butanol production.

### Effect of the mixture of glucose and glycerol on IB fermentation: toward acetone-free and 1,3-propanediol-free butanol and IB production

Although *Clostridium* sp. A1424 was shown to produce mainly butanol and isopropanol, residual acetone was still detected. Overexpression of the secondary alcohol dehydrogenase gene of *C. beijerinckii* NRRL B-593 might result in a complete conversion of acetone to isopropanol, but, according to the previous reports, *C. acetobutylicum* engineered to carry the secondary alcohol dehydrogenase gene of *C. beijerinckii* NRRL B-593 produced less butanol than the wild-type [1].

As an alternative method for no residual acetone and no negative effect on butanol production, IB fermentation of *Clostridium* sp. A1424 using mixtures of glucose and glycerol was attempted as a strategy for no residual acetone and no negative effect on butanol production. We hypothesized that the high NAD(P)H levels derived from glycerol would be advantageous for the conversion of acetone to isopropanol and, more attractively for butanol production, because NAD(P)H is required to produce those alcohols. In addition, we investigated the effect of the mixture of glucose and glycerol on 1,3-PDO production.

**Fig. 4:**
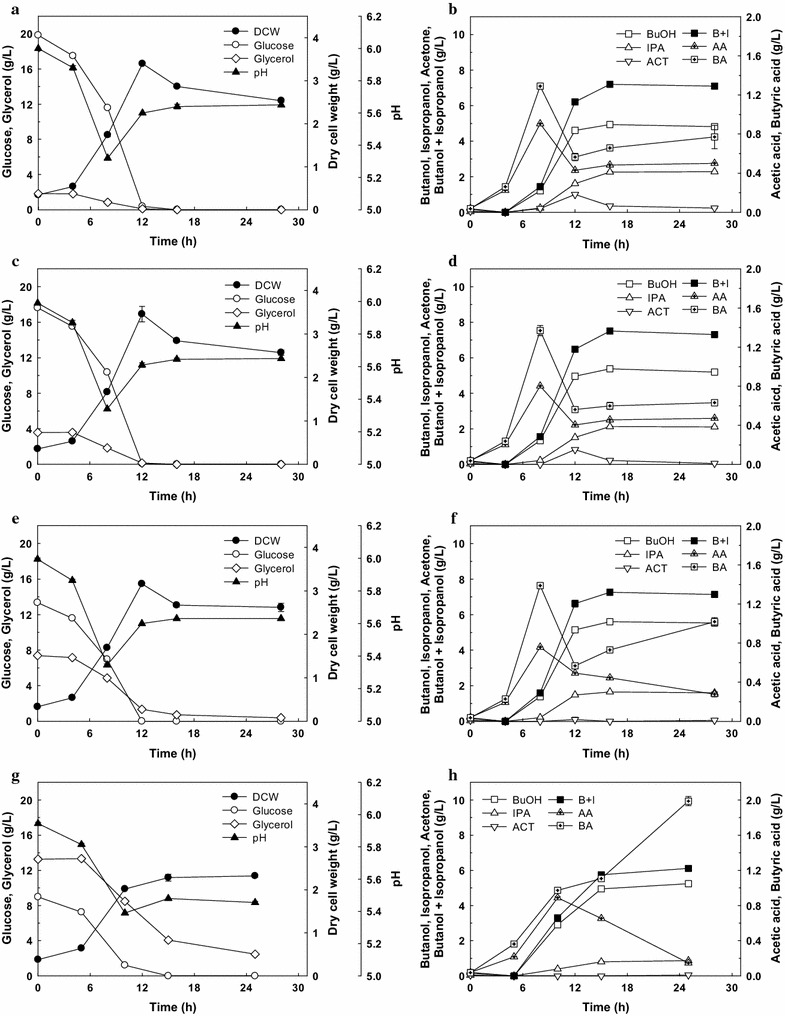
Effect of the mixture ratio of glucose and glycerol on isopropanol–butanol fermentation. The mass ratios of glucose and glycerol were 20:2 (**a**, **b**), 18:4 (**c**, **d**), 14:8 (**e**, **f**), and 9:13 (**g**, **h**). *DCW* dry cell weight, *BuOH* butanol, *IPA* isopropanol, *ACT* acetone, *B* *+* *I* butanol + isopropanol, *AA* acetic acid, *BA* butyric acid. The fermentation was performed on serum bottles without pH control. *Error bars* represent one standard deviation of triplicate experiments

To provide varied NAD(P)H generation from glycolysis, various ratios of glucose to glycerol (20:2, 18:4, 14:8, and 9:13) with a total concentration of 21–22 g/L were used. Table 3 and Fig. 4 show the effect of the ratio of glucose and glycerol on IB fermentation performance. As the ratio of glycerol increased, the total NAD(P)H from glycolysis increased (Table 3) despite the decreased total carbon consumption. The cell growth and pH profiles were not significantly different (Fig. 4a, c, e) except for the 9:13 ratio of glucose to glycerol (Fig. 4g).

**Table 3:** Isopropanol–butanol (IB) fermentation with various ratios of glucose and glycerol

	Ratio of glucose:glycerol (g:g)				
	22:0	20:2	18:4	14:8^c^	9:13^c^
Substrate consumption					
Glucose (g/L) [mM]	21.85_±0.27_ [121.25]	19.85_±0.45_ [110.16]	17.62_±0.69_ [97.78]	13.35_±0.31_ [74.10]	8.98_±0.20_ [49.82]
Glycerol (g/L) [mM]	0 [0]	1.83_±0.61_ [19.82]	3.59_±0.30_ [38.97]	7.01_±0.14_ [76.08]	10.94_±3.25_ [118.79]
Total carbon (mM)	727.53	720.43	703.59	672.83	655.27
NAD(P)H from glycolysis (mM)	242.51	259.97	273.50	300.36	337.21
NAD(P)H_C_ (mol/mol)^d^	0.33	0.36	0.39	0.45	0.51
Metabolite production					
Butanol (g/L)	4.33_±0.00_	4.66_±0.03_	5.04_±0.08_	5.38_±0.01_	5.09_±0.09_
Isopropanol (g/L)	2.44_±0.05_	2.24_±0.03_	2.07_±0.04_	1.57_±0.02_	0.83_±0.03_
Acetone (g/L)	0.28_±0.00_	0.18_±0.01_	0	0	0
Butyric acid (g/L)	0.69_±0.02_	0.73_±0.12_	0.59_±0.00_	0.98_±0.04_	1.95_±0.03_
Acetic acid (g/L)	0.51_±0.00_	0.46_±0.00_	0.43_±0.00_	0.24_±0.02_	0.11_±0.02_
NAD(P)H requirement for product (mM)	290.02	305.35	319.82	338.44	332.62
Ratio of B/I^a^ (g/g)	1.78	2.07	2.43	3.43	6.14
*Y*_B_^b^ (g/g)	0.20	0.21	0.24	0.26	0.26
*Y*_IB_^b^ (g/g)	0.31	0.32	0.34	0.34	0.30

The values are average and one standard deviation of triplicate experiments

The fermentation was performed on serum bottle without pH control

The data were obtained at 28 h of fermentation except 9:13 (25 h)

^a^B/I butanol concentration divided by isopropanol concentration

^b^ *Y*_B_, butanol yield (g butanol/g substrate); *Y*_IB_, the sum of butanol and isopropanol yield (g [butanol + isopropanol]/g substrate)

^c^After fermentations, 0.39 and 2.48 g/L glycerol remained on the ratio of 14:8 and 9:13, respectively

^d^NAD(P)H_C_: The generation of NAD(P)H mole per consumed substrate carbon mole

Regarding acetone production, when the ratio of glucose to glycerol was about 18:4 and 14:8, acetone was produced up to 0.83 and 0.10 g/L, respectively, and then completely disappeared as the fermentation was prolonged (Fig. 4d, f), clearly demonstrating the effect of glycerol addition on acetone reduction. The residual acetone was not even detected at all when the ratio of glucose to glycerol was 9:13 (Fig. 4h). Considering that *Clostridium* sp. A1424 utilizes glycerol and produces 1,3-PDO as one of the main products, it is particularly noteworthy that there was no 1,3-PDO production, even with the ratio of glucose to glycerol at 9:13. When a higher glycerol ratio (e.g., glucose:glycerol at 4:17) was examined, 1,3-PDO was produced (data not shown), indicating that adjusting the glycerol content in the mixed substrates was critical in 1,3-PDO-free IB fermentation.

Even more attractively, the concentration of butanol was higher with the mixture of glucose and glycerol (4.66–5.38 g/L) than with glucose only (4.33 g/L). The highest butanol concentration (5.38 g/L), yield of butanol (0.26 g/g), and yield of IB production (0.34 g/g) were obtained with the ratio of glucose to glycerol at 14:8 (Table 3). The productivity of butanol (*P*_B_) after 16 h was also enhanced from 0.27 g/L/h (with glucose only) to 0.35 g/L/h (glucose to glycerol ratio at 14:8). On the other hand, isopropanol production decreased as the proportion of glycerol increased (Table 3). Accordingly, the ratio of butanol to isopropanol dramatically increased from 1.78 to 6.14 with increasing proportion of glycerol (Table 3). To the best of our knowledge, this high butanol-to-isopropanol ratio has not been obtained before with wild-type or engineered clostridia strains.

**Fig. 5:**
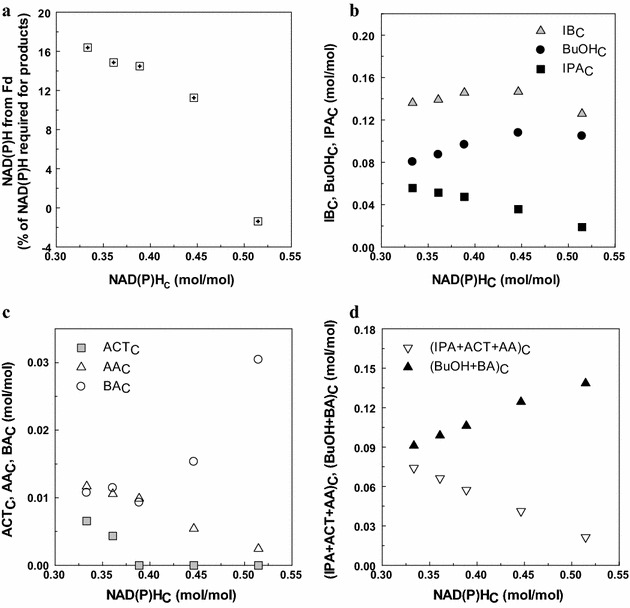
Isopropanol–butanol fermentation analysis with regard to NAD(P)H levels. A subscript “C” means ‘per C mole of consumed substrate.’ **a** Fraction of NAD(P)H from Fd per required NAD(P)H for products, **b** alcohol (IB, BuOH, and IPA) production per C mole of consumed substrate, **c** non-alcohol (ACT, AA, and BA) production per C mole of consumed substrate, **d** NAD(P)H-generating metabolites per C mole ([IPA + ACT +AA]_C_) and the other metabolites per C mole ([BuOH + BA]_C_). *BuOH* butanol, *IPA* isopropanol, *IB* isopropanol + butanol, *ACT* acetone, *AA* acetic acid, *BA* butyric acid

Further analysis of IB fermentation performance with regard to NAD(P)H levels was conducted on the basis of the moles of consumed carbon to compensate for the difference in the total carbon consumption and NAD(P)H generation capability from glucose and glycerol (0.33 and 0.66 NAD(P)H per C mole of glucose and glycerol, respectively). The NAD(P)H per C mole of consumed substrate (NAD(P)H_C_) values from the glycolysis pathway ranged from 0.36 to 0.51 mol/mol (Table 3). As seen in Fig. 5a, the fraction of NAD(P)H from Fd compared to the total NAD(P)H requirement for metabolite production decreased as the ratio of glycerol increased. Even no NAD(P)H generation from Fd seemed to be involved with the ratio of glucose to glycerol at 9:13. This indicates that increased NAD(P)H generation from glycerol likely complemented the amount of NAD(P)H required for metabolite production.

Butanol production per C mole of consumed substrate (BuOH_C_) increased with NAD(P)H_C_, but, unexpectedly, it slightly decreased at 0.51 mol/mol of NAD(P)H_C_ (i.e., glucose:glycerol = 9:13). Isopropanol per C mole of consumed substrate (IPA_C_) decreased with the increase of NAD(P)H_C_. Total alcohol production per C mole of consumed substrate (IB_C_) was the highest at the glucose to glycerol ratio of 14:8 (Fig. 5b). In cases of non-alcohol metabolites, acetone, and acetate production per C mole of consumed substrate (ACT_C_ and AA_C_, respectively) decreased with the increase of NAD(P)H_C_, while butyric acid production per C mole of consumed substrate (BA_C_) was the lowest at 0.39 mol/mol of NAD(P)H_C_. Overall, NAD(P)H_C_ at 0.45 mol/mol (glucose:glycerol = 14:8) revealed the best BuOH_C_ and IB_C_ without residual acetone, demonstrating efficient carbon conversion to butanol as well as total alcohols.

The main metabolic pathways in IB producing clostridia can be divided into net NAD(P)H-generating pathways such as acetate, acetone, and isopropanol producing pathways, while butyrate and butanol producing pathways are net NAD(P)H-neutral or net NAD(P)H-consuming ones. As seen in Fig. 5d, the sum of net NAD(P)H-generating metabolites per C mole ([ACT + IPA + AA]_C_) decreased with increasing NAD(P)H_C_ levels. On the other hand, the sum of butyric acid and butanol production per C mole ([BuOH + BA]_C_) increased with NAD(P)H_C_ levels (Fig. 5d). This result suggests that a metabolic shift likely occurred toward butyric acid and butanol production rather than acetic acid, acetone, and isopropanol production to adjust the NAD(P)H/NAD(P)^+^ balance. In Fig. 5b, c, the carbon flow to butyric acid, not butanol, increased with NAD(P)H_C_ at 0.51 mol/mol although more NAD(P)H can be consumed through butanol production. This phenomenon can be explained with a very low carbon flux to acetone and isopropanol, which likely results in a relatively low level of butyric acid re-assimilation and consequently high butyric acid residual.

The strategy using glucose and a more reduced carbon source like glycerol has been studied for the control of the metabolic fluxes and NAD(P)H pools using *C. acetobutylicum* [36] and *Clostridium butyricum* [37]. The use of mixture of glucose and glycerol has also been investigated with *C. pasteurianum* to enhance butanol production [16, 17] and with *C. beijerinckii* NCIMB 8052 for in situ detoxification of furfural [27]. However, 1,3-PDO and acetone were still produced by *C. pasteurianum* and *C. beijerinckii*, respectively, grown on the mixture of glucose and glycerol. In this study, we investigated the effect of mixture of glucose and glycerol on IB production for the first time. Notably, using a mixture of glucose and glycerol allowed us to achieve acetone-free and 1,3-PDO-free butanol and IB production with *Clostridium* sp. A1424. More desirably, the fermentation of *Clostridium* sp. A1424 with glucose and glycerol together was highly efficient for the production of butanol and total alcohol (the sum of butanol and isopropanol) along with high butanol contents. This is the first report of successful increase of butanol content in IB fermentation by using a mixture of glucose and glycerol without genetic engineering.

### High production of butanol using mixtures of glucose and crude glycerol

Because biodiesel-derived crude glycerol is an attractive cheap resource, we carried out IB fermentation with the mixture of glucose and crude glycerol instead of pure glycerol. Total substrate concentration was about 35.68 g/L with the ratio of glucose to crude glycerol at 14:8. This ratio of mixed substrates yielded the highest butanol production in case of pure glycerol (Table 3).

**Fig. 6:**
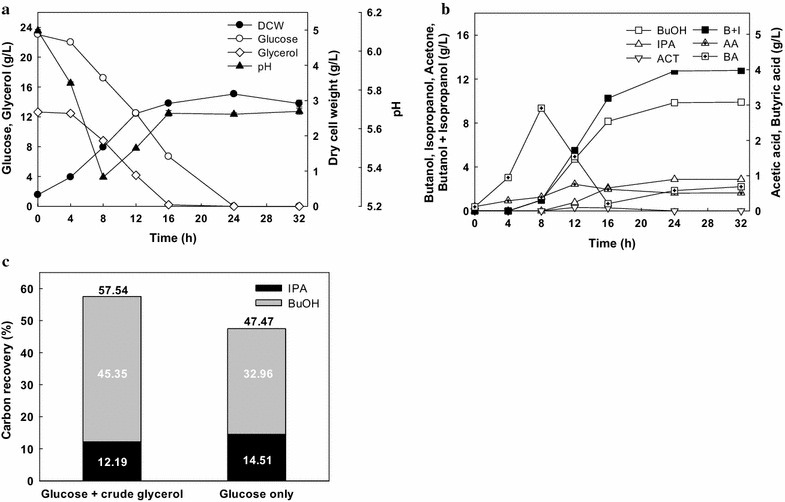
Effect of the mixture of glucose and crude glycerol on isopropanol–butanol fermentation. **a** cell growth, glucose, glycerol, and pH, **b** metabolite production, **c** carbon recovery to butanol and isopropanol. *DCW* dry cell weight, *BuOH* butanol, *IPA* isopropanol, *ACT* acetone, *B* *+* *I* butanol + isopropanol, *AA* acetic acid, *BA* butyric acid. The fermentation was performed on serum bottles without pH control. *Error bars* represent one standard deviation of triplicate experiments

As shown in Fig. 6, glucose (23.04 g/L) and crude glycerol (12.64 g/L) were completely consumed within 24 h and 12.74 g/L alcohol (9.86 g/L butanol and 2.88 g/L isopropanol) was produced. Especially, acetone-free and 1,3-PDO-free IB fermentation was also achieved using the mixture of glucose and crude glycerol. The yields of butanol and total alcohol production per total consumed substrate were 0.28 and 0.36 g/g, respectively, and the ratio of butanol to isopropanol was 3.44. These values were almost identical to those with the ratio of glucose to pure glycerol at 14:8. Therefore, crude glycerol could be used as a cost-effective co-substrate on IB fermentation by *Clostridium* sp. A1424 without inhibitory effects of impurities in crude glycerol.

Note that the concentration of butanol from the mixed substrates (9.86 g butanol/L after 24 h) and that from glucose only at pH 5.5 (9.43 g butanol/L after 32 h) (Fig. 3) are similar; but, the butanol yield and productivity using the mixed substrates are 1.4-fold higher than those with glucose only at pH 5.5 (0.29 vs. 0.20 g/g; 0.41 vs. 0.29 g/L/h). In addition, carbon recovery of substrate to butanol and IB is also significantly effective with the mixed substrates. Figure 6c shows carbon recovery of substrate to butanol and isopropanol in case of; (i) IB fermentation with the mixture of glucose and crude glycerol (the ratio of 14:8) and (ii) IB fermentation with glucose only at pH 5.5. Carbon recovery to IB was 57.5 and 47.5% with the mixed substrates and glucose only, respectively. Notably, carbon recovery to butanol with the mixed substrates was also much higher than that with glucose (45.4 vs. 33.0%). This is advantageous from the perspective of carbon recovery and carbon conversion to bio-fuel (butanol). Further study, such as the development of genetic tools and process optimization would be helpful for achieving improved yield, productivity, and concentration for IB fermentation.

## Conclusions

A newly isolated *Clostridium* sp. A1424 was able to produce not only butanol and isopropanol from various carbon sources including glucose, but also butanol from glycerol as the main product. The productivity and titer of butanol and total alcohol with *Clostridium* sp. A1424 were higher than the previous reports exploring other natural IB producers. Use of the mixture of glucose and glycerol (regardless of pure and crude glycerol) was successful to achieve acetone-free, 1,3-PDO-free, and enhanced IB production. Particularly, the performance of butanol production (yield, productivity, and selectivity) was significantly enhanced using a mixture of glucose and glycerol, compared to using glucose only. Thus, IB fermentation favoring butanol production with *Clostridium* sp. A1424 is especially valuable for the efficient carbon recovery to IB and butanol, a promising biofuel.

## Methods

### Media

Modified P2 medium (MP2) was used for seed and main cultures. The composition of medium (wt/v) was 0.6% yeast extract, 0.05% K_2_HPO_4_, 0.05% KH_2_PO_4_, 0.2% (NH_4_)_2_SO_4_, 0.02% MgSO_4_·7H_2_O, 0.001% MnSO_4_·H_2_O, 0.001% FeSO_4_·7H_2_O, 0.001% NaCl, and 1.95% MES (2-(*N*-morpholino) ethanesulfonic acid) [12]. Various carbon sources including d-glucose, d-mannose, d-fructose, d-xylose, l-arabinose, d-galactose, d-cellobiose, sucrose, and glycerol were added to MP2 medium as a substrate at a certain concentration as occasion demanded. Crude glycerol containing 81.7% (wt/wt) of glycerol, 10.5% (wt/wt) of water, 5% (wt/wt) of MONG (matter organic non-glycerol), 2.9% (wt/wt) of ash, 2.4% (wt/wt) of sodium, and less than 0.01% (wt/wt) of methanol, magnesium, and potassium was obtained from GS Caltex Corporation (South Korea) [38]. The initial pH of the medium was adjusted to 6.0 with 5 N KOH.

### Isolation and identification of *Clostridium* sp. A1424

Diesel-oil-contaminated soil was collected from Baegun Mountain (Gyeonggi-Do, South Korea). The soil sample (2 g) was added to 20 mL of sterilized and anaerobic MP2 medium containing 2% (wt/v) glucose; then heated at 80 °C for 30 min. The solution was incubated at 30 °C until cell growth was observed. After confirming the cell growth with the optical density at 600 nm (OD_600_), the culture broth was serially diluted with 0.85% (wt/v) sterilized saline solution. Then it was plated onto MP2 medium agar plate containing 2% (wt/v) glucose in an anaerobic chamber (85% N_2_, 10% CO_2_, and 5% H_2_; Coy Laboratory Products, Ann Arbor, MI, USA). After the cultivation for 4 days at 30 °C in the chamber, each colony was cultivated again on MP2 liquid medium with 2% (wt/v) glucose for 48 h and metabolites were analyzed.

The 16S rRNA analysis and phylogenetic analysis of *Clostridium* sp. A1424 were accomplished as described in Lee et al. [38]. The 16S rRNA sequence was deposited to GenBank with the accession number KT314078 (http://www.ncbi.nlm.nih.gov).

### Culture conditions

The microorganism was stored as a spore solution in sterile distilled water at 4 °C. For the activation of spores, the solution was heated at 80 °C for 10 min. The seed cultivation was started with inoculation of the 5% (v/v) activated spore solution in autoclaved MP2 medium containing 2% (wt/v) glucose at 37 °C and 200 rpm. When cell growth reached OD_600_ of 8.0–9.0 in the late-exponential phase, 5% (v/v) culture solution was used to inoculate the main culture medium. Then, the main cultivation was performed at 37 °C and 200 rpm. To achieve anaerobic conditions for the seed and main cultures, the medium in the serum bottle was purged with argon gas for 20 min and the bottle was sealed with a butyl stopper and aluminum crimp seal [38].

For the pH-controlled fermentations, a 3-L bench fermenter (Fermentec, South Korea) with a working volume of 1 L was prepared. The MP2 medium containing 6% (wt/v) glucose without MES was used. The fermenter jar was autoclaved and purged with filtered argon gas for 1 h to establish anaerobic conditions. Then, the 5% (v/v) seed culture solution was inoculated. The fermentation was operated at 37 °C and 200 rpm and the pH was controlled with 5 N KOH and HCl at the set point (pH 5.0–6.0). In case of bubble formation on the surface of the culture broth, 25% (v/v) anti-foam solution (Antifoam Y-30 emulsion, Sigma Chemical Co., MO, USA) was added intermittently.

### Analysis and calculation

Cell growth was monitored with OD at 600 nm using a spectrophotometer (Shimadzu UVmini-1240 spectrophotometer, Kyoto, Japan) and dry cell weight (DCW, g/L) was determined using a calibration curve of OD_600_ and DCW. The amount of carbon sources (except sucrose), 1,3-PDO, acetic acid, and butyric acid were analyzed by HPLC (Agilent technology 1,200 series, CA, USA) composed of a refractive index detector (RID) and UV/Vis detector with a Hi-plex H column (300 × 7.7 mm, Agilent technology, CA, USA). A mobile phase was 5 mM sulfuric acid with a flow rate of 0.6 mL/min. Sucrose content was measured using a sucrose test kit (Merck Millipore Co., Darmstadt, Germany). Acetone, isopropanol, ethanol, and butanol were analyzed using a GC (Agilent technology 6890N, CA, USA) equipped with a flame ionization detector (FID). A DB-624 column (0.53 mm × 30 m × 3.0 μm, Agilent technology, CA, USA) was used to clearly separate each peak of ethanol and isopropanol. The production of ethanol was excluded as the detected concentration was negligible (below 0.1 g/L) in all experiments.

The generation of NAD(P)H from glucose and glycerol through the glycolytic pathway was calculated by multiplying ‘2’ by the moles of consumed glucose and glycerol, respectively. The requirement of NAD(P)H for the production was calculated by multiplying 4, 2, 1, 0, and 0 by the number of moles of produced butanol, butyric acid, isopropanol, acetone, and acetic acid, respectively [12] (Additional file 3: Table S1).

## Additional files

**Additional file 1: Figure S1.** The fermentation profile with xylose as a sole carbon source. (a) Cell growth, substrate consumption, and pH, (b) products. DCW, dry cell weight; BuOH, butanol; IPA, isopropanol; ACT, acetone; AA, acetic acid; BA, butyric acid. Error bars represent one standard deviation of triplicate experiments.

**Additional file 2: Figure S2.** The fermentation profile using glucose (a and b) and xylose (c and d) as a sole carbon source with acetone in media. DCW, dry cell weight; BuOH, butanol; IPA, isopropanol; ACT, acetone.

**Additional file 3: Table S1.** Net NADH balance per one mole of product formation from the corresponding mole of glucose and glycerol.

**Additional file 4: Figure S3.** Calculated NAD(P)H balance between substrates and products. The molar concentration of NAD(P)H from Fd_red_ was calculated as following: *M*_NAD(P)H from Fd_ = 4 × *M*_Butanol_ + 1 × *M*_Isopropanol_ + 2 × *M*_Butyric acid_ − 2 × *M*_Glucose._

## Abbreviations

1,3-PDO: 1,3-propanediol
IBE: mixture of isopropanol, butanol, and ethanol
ABE: mixture of acetone, butanol, and ethanol
PFOR: pyruvate ferredoxin oxidoreductase
Fd: ferredoxin
Fd_red_: reduced ferredoxin
MP2: modified P2 medium
MONG: matter organic non-glycerol
OD_600_: optical density at 600 nm
