# Supplementary material for: Effective isopropanol–butanol (IB) fermentation with high butanol content using a newly isolated Clostridium sp. A1424
Source: Biotechnol Biofuels. 2016 Oct 26;9:230. doi: 10.1186/s13068-016-0650-7 (PMC5080687; doi:10.1186/s13068-016-0650-7)
Supplement: Supplementary file 1 — Additional file 1: Figure S1. The fermentation profile with xylose as a sole carbon source. (a) Cell growth, substrate consumption, and pH, (b) products. DCW, dry cell weight; BuOH, butanol; IPA, isopropanol; ACT, acetone; AA, acetic acid; BA, butyric acid. Error bars represent one standard deviation of triplicate experiments. [file 13068_2016_650_MOESM1_ESM.pdf]

Supplementary information

a 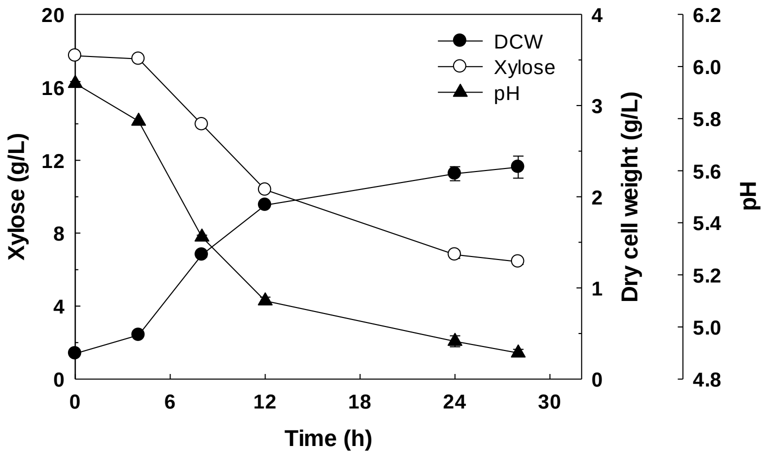

b 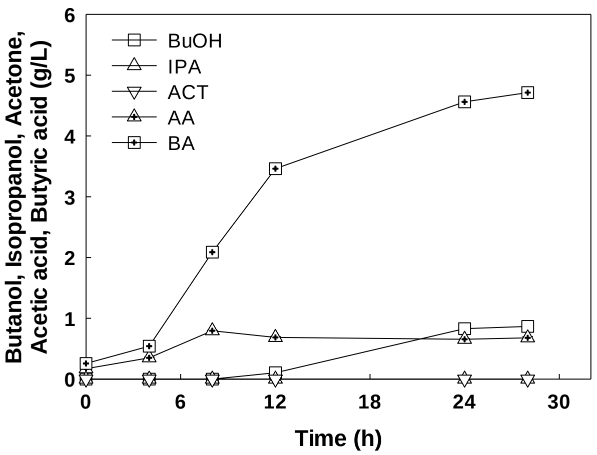

Figure. S1. The fermentation profile with xylose as a sole carbon source. (a) Cell growth, substrate consumption, and pH, (b) products. DCW, dry cell weight; BuOH, butanol; IPA, isopropanol; ACT, acetone; AA, acetic acid; BA, butyric acid.

All results are averages of triplicate experiments.
