# Supplementary material for: Effective isopropanol–butanol (IB) fermentation with high butanol content using a newly isolated Clostridium sp. A1424
Source: Biotechnol Biofuels. 2016 Oct 26;9:230. doi: 10.1186/s13068-016-0650-7 (PMC5080687; doi:10.1186/s13068-016-0650-7)
Supplement: Supplementary file 2 — Additional file 2: Figure S2. The fermentation profile using glucose (a and b) and xylose (c and d) as a sole carbon source with acetone in media. DCW, dry cell weight; BuOH, butanol; IPA, isopropanol; ACT, acetone. [file 13068_2016_650_MOESM2_ESM.pdf]

Supplementary information

Figure S2.

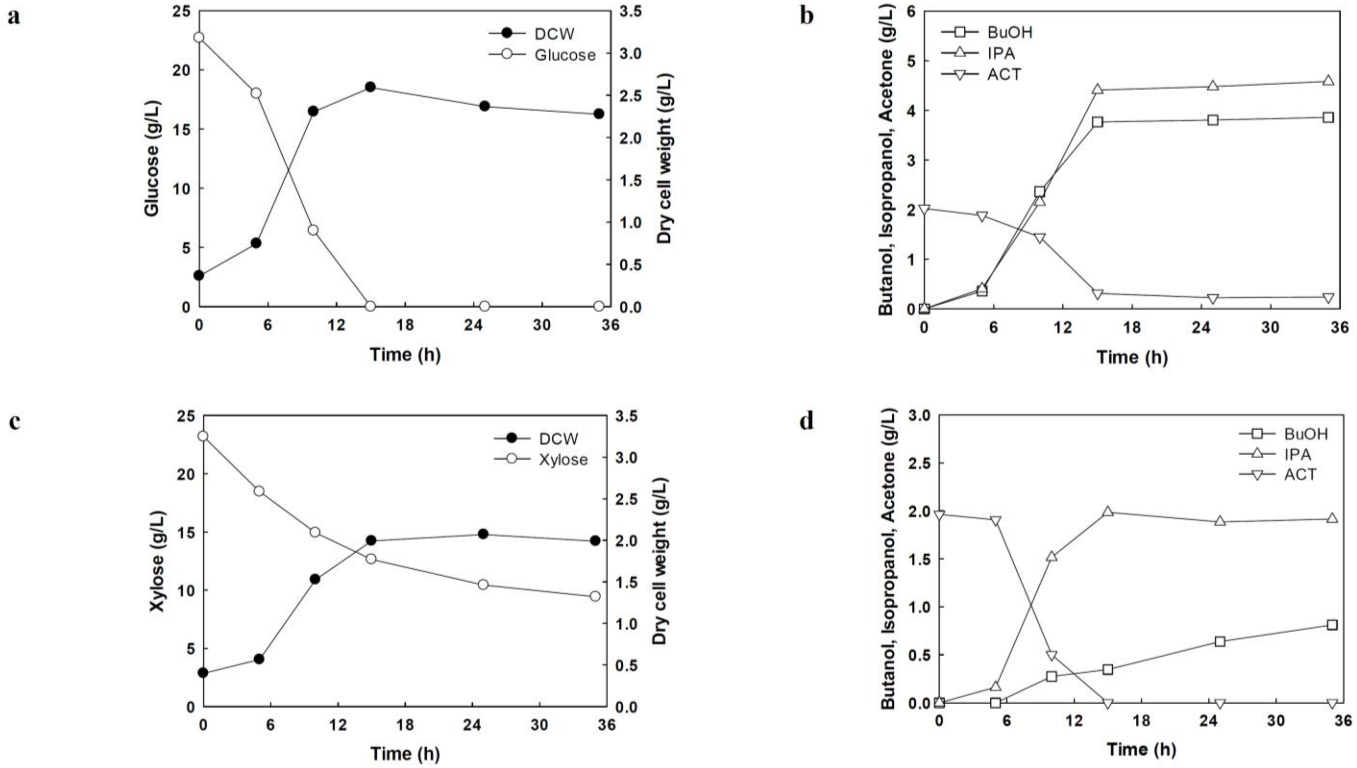

Figure S2. The fermentation profile using glucose (a and b) and xylose (c and d) as a sole carbon source with acetone in media. DCW, dry cell weight; BuOH, butanol; IPA, isopropanol; ACT, acetone
