# Supplementary material for: Effective isopropanol–butanol (IB) fermentation with high butanol content using a newly isolated Clostridium sp. A1424
Source: Biotechnol Biofuels. 2016 Oct 26;9:230. doi: 10.1186/s13068-016-0650-7 (PMC5080687; doi:10.1186/s13068-016-0650-7)
Supplement: Supplementary file 3 — Additional file 3: Table S1. Net NADH balance per one mole of product formation from the corresponding mole of glucose and glycerol. [file 13068_2016_650_MOESM3_ESM.pdf]

# Supplementary information

Table S1 Net NADH balance per one mole of product formation from the corresponding mole of glucose and glycerol.

| Product | From glucose | | | From glycerol | | |
|---|---|---|---|---|---|---|
| | Generated | Consumed | Net | Generated | Consumed | Net |
| Butanol | +2 NAD(P)H | -4 NAD(P)H | -2 | +4 NAD(P)H | -4 NAD(P)H | 0 |
| Isopropanol | +2 NAD(P)H | -1 NAD(P)H | +1 | +4 NAD(P)H | -1 NAD(P)H | +3 |
| Acetone | +2 NAD(P)H | 0 NAD(P)H | +2 | +4 NAD(P)H | 0 NAD(P)H | +4 |
| Butyrate | +2 NADH | - 2 NADH | 0 | +4 NADH | -2 NADH | +2 |
| Acetate | +1 NADH | 0 NADH | +1 | +2 NADH | 0 NADH | +2 |
