# Supplementary material for: Effective isopropanol–butanol (IB) fermentation with high butanol content using a newly isolated Clostridium sp. A1424
Source: Biotechnol Biofuels. 2016 Oct 26;9:230. doi: 10.1186/s13068-016-0650-7 (PMC5080687; doi:10.1186/s13068-016-0650-7)
Supplement: Supplementary file 4 — Additional file 4: Figure S3. Calculated NAD(P)H balance between substrates and products. The molar concentration of NAD(P)H from Fdred was calculated as following: M NAD(P)H from Fd = 4 × M Butanol + 1 × M Isopropanol + 2 × M Butyric acid − 2 × M Glucose. [file 13068_2016_650_MOESM4_ESM.pdf]

Supporting information

Figure S3.

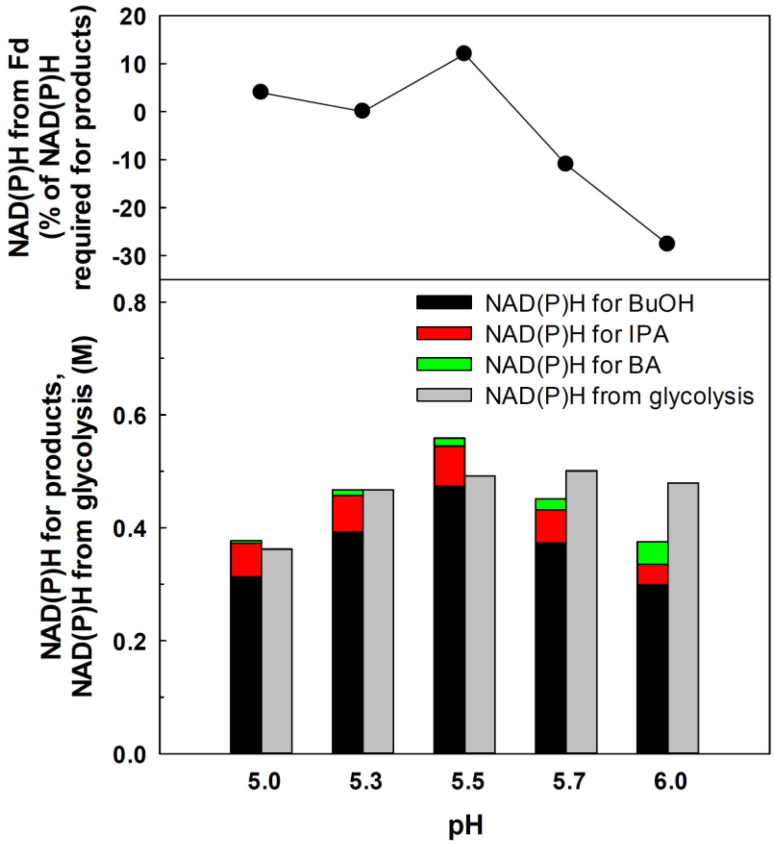

Figure S3. Calculated NAD(P)H balance between substrates and products. The molar concentration of NAD(P)H from Fd_{red} was calculated as following:

$M_{\mathrm{NAD(P)H\ from\ Fd}} = 4\times M_{\mathrm{Butanol}} + 1\times M_{\mathrm{Isopropanol}} + 2\times M_{\mathrm{Butyric\ acid}} - 2\times M_{\mathrm{Glucose}}$
